# *Colletotrichum* species associated with jute (*Corchorus capsularis* L.) anthracnose in southeastern China

**DOI:** 10.1038/srep25179

**Published:** 2016-04-28

**Authors:** Xiaoping Niu, Hong Gao, Jianmin Qi, Miancai Chen, Aifen Tao, Jiantang Xu, Zhigang Dai, Jianguang Su

**Affiliations:** 1 Key Laboratory for Genetics, Breeding and Multiple Utilization of Crops, Fujian Agriculture and Forestry University, Fuzhou 350002, PR China; 2 Key Laboratory for Control of Plant Diseases and Insect Pests, Institute of Environment & Plant Protection, Hainan Academy of Agricultural Sciences, Haikou 571100, China; 3 Institute of Bast Fiber Crops, Chinese Academy of Agricultural Sciences, Changsha 410205, China

## Abstract

Anthracnose, caused by the *Colletotrichum* species of fungi, is one of the most serious diseases affecting jute in China. The disease causes chlorotic regions with black brown sunken necrotic pits on the surfaces of stems. In late stages of disease, plants undergo defoliation, dieback and blight, which make anthracnose a major threat to jute fiber production and quality in China. In this study, 7 strains of *Colletotrichum* fungi were isolated from diseased jute stems from Zhejiang, Fujian, Guangxi, and Henan plantations in China. Multi-locus sequence (ACT, TUB2, CAL, GS, GAPDH and ITS) analysis coupled with morphological assessment revealed that *C. fructicola*, *C. siamense* and *C. corchorum-capsularis* sp. nov. were associated with jute anthracnose in southeastern China. *C. fructicola* and *C. siamense* were previously not associated with jute anthracnose. *C. corchorum-capsularis* is a new species formally described here. Pathogenicity tests confirmed that all species can infect jute, causing anthracnose, however the virulence of the 3 species differed. This report is the first associating these three species with jute disease worldwide and is the first description of the pathogens responsible for jute anthracnose in China.

Jute (*Corchorus capsularis* L.) is an annual bast fiber crop. It is found predominantly in Southeast Asia After cotton, jute is the second cheapest and second most commercially available fiber crop, making it an abundant source of biodegradable and renewable lignocellulose fiber^1^. Due in large part to its high luster, moisture absorption properties, ability for rapid water loss, and easy degradation, jute fibers have been exploited in various value-added products such as flooring and textiles^2^. However, jute is affected by a variety of diseases at all stages of its development, from seed germination to the harvested fruits. Anthracnose, caused by *Colletotrichum* species, has recently become the most serious disease of jute in China. This disease results in sunken necrotic lesions on the surfaces of stems that limit fiber productivity and reduce fiber quality.

Many species of the fungal genera *Colletotrichum* cause a variety of diseases in a wide range of economically important plants around the world^3^,^4^,^5^. Previously, the identification of *Colletotrichum* species was based on morphological characteristics^3^. Cai *et al.* and Cannon *et al.* found that such morphological identifications of the species of *Colletotrichum* depended on experimental methods used, which caused the taxonomy and nomenclature to be inconsistent^4^,^6^,^7^. Recently, Cai *et al.* recommended a polyphasic approach for accurate identification of *Colletotrichum* species using multi-locus phylogeny coupled with morphological data^6^,^8^. Using this approach, many *Colletotrichum* strains have been successfully identified and epitypified^9^,^10^,^11^,^12^,^13^,^14^,^15^,^16^. This increased understanding of *Colletotrichum* species can increase the effectiveness of plant disease control interventions^7^,^8^,^14^,^17^.

Prior to the polyphasic identification of *Colletotrichum* species, *C. gloeosporioides* and *C. corchorum* were generally recognized as the most important jute pathogens worldwide^18^,^19^. However, these identifications were based on inadequate techniques including examination of plant symptoms, assessment of the morphology of conidia produced on the infected tissues, or morphology on potato dextrose agar (PDA) cultures. Additionally, following the epitypification of *C. gloeosporioides*^20^, Phoulivong *et al.* reported that *C. gloeosporioides* sensu stricto was, in fact, not a common pathogen in the tropics^21^. In China, anthracnose of jute is attributed exclusively to the species *C. gloeosporioides* and *C. corchorum*, however there are no studies that perform molecular characterization of *Colletotrichum* species on jute. Therefore, this study was conducted to unambiguously identify the species of *Colletotrichum* that cause jute anthracnose by combining morphological and molecular approaches. Further, we aimed to determine the pathogenicity and distribution of the *Colletotrichum* species associated with jute anthracnose in China.

## Materials and Methods

### Sampling and spore isolation

From 2011–2012, jute stems showing symptoms of anthracnose were collected from plantations located in Zhejiang, Fujian, Guangxi and Henan provinces of China. 3 pieces (5 × 5 mm) of stem tissue from each plant were surface sterilized in 70% ethanol for 45 s followed by 1% NaClO for 1 min. Samples were then rinsed three times with sterilized water and dried on sterile tissue paper. Samples were placed on PDA and incubated at 25 °C for 2–4 days. Additionally, the leading edge of any fungal hyphae that grew from the tissues was transferred aseptically to PDA. Fungi were monitored for sporulation and spore masses were picked off with a sterilized wire loop and streaked on the surface of water agar. After incubation overnight at 25 °C, single germinated spores were picked up with a sterile needle and transferred to PDA^13^. Using the procedure described by Cai, *et al.*, single spore cultures were obtained for each *Colletotrichum* isolate. These pure cultures were stored in sterilized water in Eppendorf tubes at 4 °C and stock cultures were stored in PDA slants at 4 °C in the dark.

### Morphological studies of Colletotrichum from jute

Referred to the method described by Cai, *et al.*, characterization of spore morphology and growth in culture were performed^6^. Mycelial discs (5 mm diameter) were taken from actively sporulating areas near the growing edge of cultures after 5 days of growth and transferred to PDA. Three replicate cultures of each isolate were incubated at 25 °C in the dark. After 7 days, colony diameter was measured and growth rate was calculated as the total growth divided by seven. Colony characteristics of conidial masses and zonation were also recorded^6^,^15^.

Appressoria were obtained by use of a slide culture technique in which 1 cm^2^ square of agar was inoculated on one side with conidia and then covered with a sterile cover slip^6^. The shape and size of the appressoria formed across the underside of the cover slip were studied after 5–7 days of incubation at 25 °C. Morphological data were analyzed using analysis of variance (P < 0.05) with Duncan’s Test.

### DNA extraction, PCR amplification and Sequencing

Isolates were grown on PDA and incubated at 25 °C for 7 days. Mycelium was scraped from the colony surface using a sterile 10 μl pipette tip. Genomic DNA was extracted from the mycelium using the Biospin Fungus Genomic DNA Extraction Kit (BioFlux®) according to the manufacturer’s protocol. DNA concentrations were estimated visually on a 1% agarose gel by comparing band intensity with a 1 kb DNA ladder (Transgen Biotech®).

Partial actin (ACT), calmodulin (CAL), β-tubulin (TUB2), glutamine synthetase (GS), glyceraldehyde-3-phosphate dehydrogenase (GPDH) genes and the complete rDNA-ITS (ITS) region from 7 *Colletotrichum* strains were amplified by PCR. Primer pairs for PCR amplifications were referred to the method described by Prihastuti, *et al.* The PCR products were examined by electrophoresis in 1% agarose gels, purified, and ligated into the pMD18-T vector (Takara, Japan). The vectors containing these gene fragments were transformed into *Escherichia coli* DH5α and DNA sequencing was performed by BGI Company, Shanghai, China. Sequences derived in this study are deposited in GenBank. The accession numbers of all sequences analyzed in this study are listed in Table 1.

### Phylogenetic analysis

Sequences from our isolates, together with reference sequences obtained from GenBank (Table 1), were aligned using ClustalW in MEGA v.5^22^. The multi-locus dataset was subsequently aligned using MAFFT v.6^23^, and manually adjusted using Notepad+ +when necessary. A maximum parsimony (MP) analysis was performed on the multi-locus alignment (ACT, CAL, GAPDH, GS, ITS, TUB2) using PAPU v.4.0b10^24^. All ambiguously aligned regions were excluded from analyses. Unweighted parsimony (UP) analysis was performed. Trees were inferred using the heuristic search option with Tree Bisection Reconnection (TBR) branch swapping and 1000 random sequence additions. Maxtrees were unlimited, branches of zero length were collapsed and all multiple parsimony trees were saved. Descriptive tree statistics were recorded, including tree length (TL), consistency index (CI), retention index (RI), rescaled consistency index (RC), and homoplasy index (HI). Robustness of clades was assessed by a bootstrap analysis with 1000 replicates.

In addition, the Markov Chain Monte Carlo (MCMC) algorithm was used to regenerate the phylogenetic trees with Bayesian posterior probabilities in MrBayes v.3.2.1^25^. MrModeltest v.2.3^26^ was used to determine statistical selection of best-fit models of nucleotide substitutions. Two analyses of four MCMC chains were run from random trees for 10 million generations and sampled every 1000 generations. The first 25% of trees generated were discarded because they represented the burn-in phase of each analysis. The remaining trees were used for calculating the posterior probabilities in the majority rule consensus tree.

2 isolates were used in the initial MP analysis using a concatenated alignment for 4 genes: CAL, GAPDH, GS and TUB2. *Colletotrichum boninense* (MAFF 305972) was used as outgroup in this analysis. A second analysis was carried out to confirm the identity of five isolates with curved conidia based on a concatenated alignment of 6 genes: ACT, CAL, GAPDH, GS, ITS and TUB2. *Colletotrichum lindemuthianum* (CBS 151.28) was used as the outgroup in this second analysis. Phylogenetic trees were created in Figtree^27^.

### Pathogenicity tests

The isolates that were characterized by morphology were also submitted to pathogenicity tests. Single spore isolates were incubated on PDA for 7 days at 28 °C. Conidial suspensions were prepared by adding 10 ml of sterile distilled water to the culture, swirling to isolate the conidia, and filtering through two layers of muslin cloth. Spore concentration was adjusted to 10^6^ conidia/ml with sterile water using a hemocytometer. Jute leaves and stems without symptoms of disease were washed with tap water, surface disinfected in 75% ethanol for 60 sec and 1% sodium hypochlorite for 5 min, and then washed 3 times with sterile distilled water and dried in a fume hood. Spore suspensions, or sterile water for the negative control, were sprayed on the jute leaves. Stems were inoculated by using a sterile scalpel to create superficial wounds in the stem epidermis. The wound was then inoculated with a 5-mm-diameter PDA disk selected from the edge of an actively growing culture. Stems inoculated with sterile PDA were used as a negative control. The inoculated plants were kept in plastic containers, covered with plastic wrap to maintain humidity, and incubated at 28 °C with 12/12 h fluorescent light and darkness. 10 jute seedlings were inoculated for each species; the experiment was performed in triplicate. The incidence of infection was calculated by the formula [Incidence (%) = (infected sites or leaves/inoculated sites or leaves) × 100%] at 12-days post inoculation. The incidence data was analyzed using SPSS software version 20.0 (SPSS Inc., Chicago, USA).

## Results

### Collection of Colletotrichum species

In total, 7 *Colletotrichum* strains were isolated from diseased jute samples from the main jute growing regions (Zhejiang, Fujian, Guangxi and Henan provinces) of China. Based on the morphological characterization on PDA, 2 strains produced conidia similar to *C. gloeosporioides*. 5 strains produced curved conidia, which is typical of fungi in the *C. truncatun* species complex^28^.

### Phylogenetic analysis

**Figure 1:**
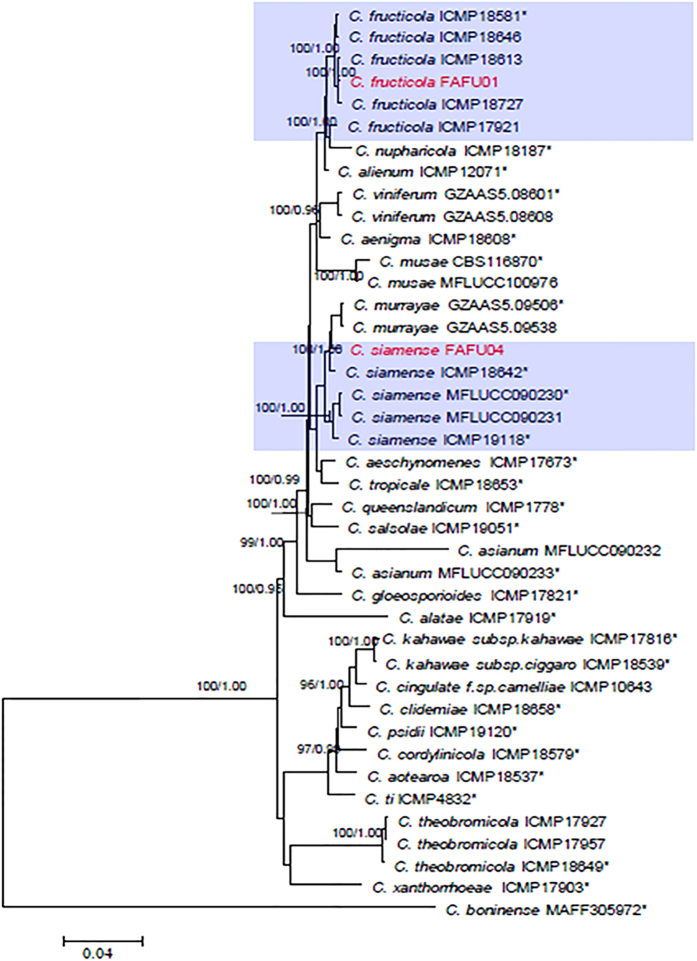
Maximum parsimony tree obtained from a heuristic search of the combined CAL, GAPDH, GS and TUB2 sequence alignment. Bootstrap support values ≥ 50% and Bayesian posterior probability values ≥ 0.5 are shown at the nodes. *C. boninense* was used as the outgroup. * indicates the ex-type strains. Strains isolated in this study are shown in red.

**Figure 2:**
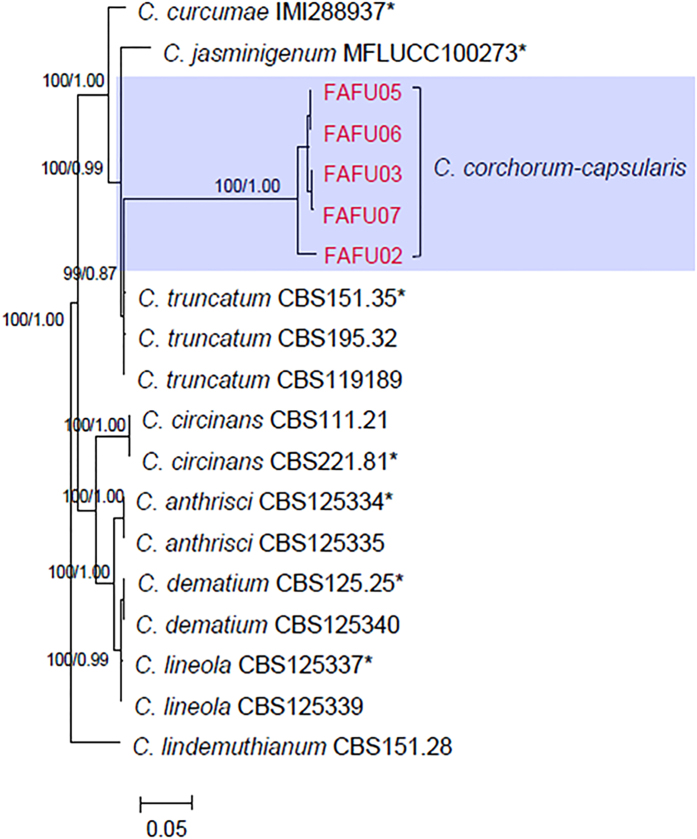
Maximum parsimony tree obtained from a heuristic search of the combined ACT, CAL, GAPDH, GS, ITS and TUB2 sequence alignments, showing the phylogenetic relationships of *Colletotrichum* species isolated from *C. corchorum-capsularis*. Bootstrap support values ≥ 50% and Bayesian posterior probability values ≥ 0.5 are shown at the nodes. *C*. *lindemuthianum* was used as the outgroup. * indicates the ex-type strains. Strains isolated in this study are shown in red.

Molecular analyses were performed on all of the *Colletotrichum* strains isolated, including 2 strains from the *C. gloeosporioides* complex and 5 strains with curved conidia. Figure 1 shows the phylogram constructed to identify the strains in the *C. gloeosporioides* species complex. The strain, FAFU01, could be confidently identified as *C. fructicola* as it clustered together with the ex-epitype strain ICMP 18581 with 100% bootstrap support. Another strain, FAFU04, clustered with *C. siamense* strains with 100% bootstrap support, based on the combined datasets of partial CAL, GAPDH, GS and TUB2 sequence analysis. The other 5 strains did not cluster with any currently known species based on these 4 molecular markers. Therefore, a further 6 gene regions (ACT, CAL, GAPDH, GS, ITS and TUB2) of these five strains were sequenced and phylogenetic relationships were predicted using parsimony and Bayesian methods (Fig. 2). However, these 5 strains did not cluster well with any other *Colletotrichum* species in the 6 gene phylogenetic tree (Fig. 2). The morphological and culture characteristics were closest to the species *C. corchorum* previously reported^18^, so these 5 strains are described herein as *C. corchorum-capsularis* sp. nov.

### Taxonomy

#### Colletotrichum corchorum-capsularis

**Figure 3:**
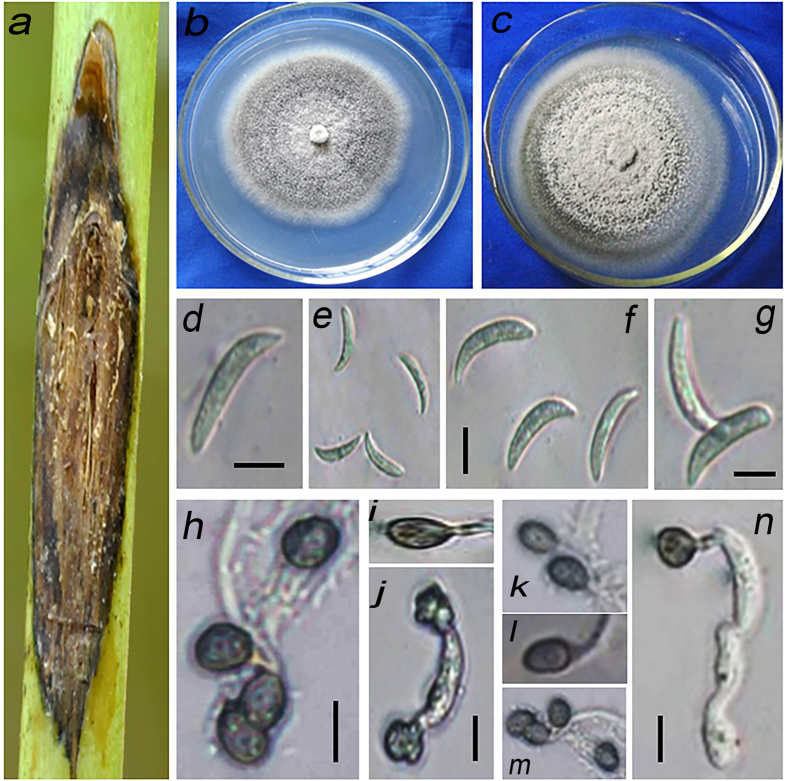
*C. corchorum-capsularis* (FAFU02). (**a)** Symptom of anthracnose on the stem of jute. (**b,c)**. Colony on PDA of different isolates of FAFU02. (**d–g)** Conidia. (**h–n)** Appressoria. Scale: (**d–g**) = 2 μm; (**h**–**n**) = 10 μm.

Xiaoping Niu, Hong Gao, Jianmin Qi, Miancai Chen and Jianguang Su, sp. nov. Fig. 3.

Fungal Names: FN570235.

#### Etymology

Named after its host, *Corchorus capsularis*.

When inoculated on PDA, colonies grew 6.5–10.5 mm/day in diameter at 28 °C. After 7 days, isolates with greyish white to dark gray mycelium and dense, concentric, circular conidia masses were observed.

#### Conidia

18.3–26.3 × 2.7–4.3 μm (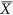  = 22.6 × 3.62 μm), hyaline, non-septate, smooth walled, curved, falcate-fusoid. Appressoria: 6.8–12.5 × 6.0–9.8 μm (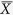 = 8.48 × 7.26 μm) diam, produced from mycelia, brown, ovoid to ellipsoidal. Sexual state was not observed (Table 2; Fig. 3).

Host: FAFU02, FAFU03, FAFU05, FAFU06 and FAFU07 were isolated from the stems of jute (*Corchorus capsularis*) that was black and withered.

Known distribution: Youxi and Zhaoan, Fujian Province; Xinyang, Henan Province and Xiaoshan, Zhejiang Province, China.

Material examined: CHINA, Fujian Province, Youxi and Zhaoan; Henan Province, Xinyang; Zhejiang Province, Xiaoshan, isolated from stems of *Corchorus capsularis*, 22–28 June 2013, Xiaoping Niu and Hong Gao, type culture FAFU02.

Notes: All 5 strains form a distinct clade with 100% bootstrap support, indicating that they represent a distinct species. Referring to their colony characteristics, *C. corchorum-capsularis* is introduced to accommodate this species. This species is similar to *C. corchorum* by its morphological characteristics and growth in culture. They both produced greyish white and cottony colonies, and grew 6.5–10.5 mm/day in diameter at 28 °C. However, the conidial length was longer (18.3–26.3 μm) than that from *C. corchorum* (12.0–25.0 μm).

#### Colletotrichum fructicola

Prihastuti, H., Cai, L. & Hyde, K.D. Fungal Diversity 39:96 (2009).

Material examined: CHINA, Fujian Province, Putian, isolated from stems of *Corchorus capsularis*, 20 June 2013, Xiaoping Niu and Hong Gao, culture FAFU01 = BPD-I18.

Notes: *Colletotrichum fructicola* was originally reported as a pathogen of coffee berries in Thailand^10^. This species was also known as a pathogen of *Pyrus pyrifolia* (Japan), *Persea americana* (Australia), *Malus domestica* (Brazil), *Dioscorea* (Nigeria), *Theobroma* and *Tetragastris* (Panama)^15^, *Vitis* (China)^14^, and *Mangifera indica* (Brazil)^13^. Strain FAFU01 in our study was identified as *C. fructicola* based on morphology and multi-locus (CAL, GAPDH, GS and TUB2) phylogenetic analysis. In the phylogram, the strain clustered with *C. fructicola* (ICMP 18581) with 100% bootstrap support and posterior probability values of 1.00 (Fig. 1).

#### Colletotrichum siamense

Prihastuti, H., Cai, L. & Hyde, K.D. Fungal Diversity 39:98 (2009).

Material examined: CHINA, Guangxi Province, Nanning, cultured from stems of jute, 16 June 2013, Xiaoping Niu and Hong Gao, culture FAFU04 = BPD-I2. THAILAND, Chiang Mai, Mae Lod Village, on *Coffea arabica* berries, 12 December 2007, Prihastuti, H., culture CBS 130417 = ICMP 18642 = MFLUCC 090230 = BPD-I2.

Notes: A detailed description of *Colletotrichum siamense* was provided by Prihastuti, *et al. C. siamense* was also reported as a pathogen of *Hymenocallis* sp. (China), *Malus* (USA), *Jasminum* (Vietnam), *Dioscoria* (Nigeria), *Persea* and *Pistacia* (Australia)^15^, and *Proteaceae*^29^. In the present study, *C. siamense* was isolated from stems of jute. The conidial shape and dimensions match the holotype of *C. siamense*^10^. However, the appressoria were (4.8–9.6 μm wide) slightly wider than that from ex-holotype culture (3.5–5.3 μm wide)^10^. In the phylogram, this strain clustered together with the type strain of *C. siamense* (ICMP18642) and strain MFLUCC090230 with bootstrap support/posterior probability values of 100%/0.99 and 100%/1.00, respectively (Fig. 1).

### Pathogenicity testing

The pathogenicity of the *Colletotrichum* isolates was tested on both leaves and stems of jute to confirm Koch’s postulates. As shown in Table 3, the 3 species recovered in this study exhibited different virulence. *Colletotrichum corchorum-capsularis* strain FAFU02 was the most virulent on experimental leaves, with a mean infection incidence of 83%. *Colletotrichum fructicola* strain FAFU01 was also pathogenic to jute leaves with a mean infection incidence of 62%. *Colletotrichum siamense* strain FAFU04 infected experimental leaves with a lower mean infection incidence (58%) but this was not significantly different from strain FAFU01. As for lesion size on stems, *C. corchorum-capsularis* strain FAFU02 produced the largest lesions (mean length = 10.7 ± 1.70 mm, mean width = 6.4 ± 0.27 mm).

## Discussion

*Colletotrichum* species on jute (*C. capsularis*) have been poorly studied, with reports focusing on *C. gloeosporioides* and *C. corchorum*^18^,^19^. Previous studies on *Colletotrichum* species causing disease on jute used morphological and culture characterizations which restrains identification to species complexes rather than individual species. The current study represents the first identification of *Colletotrichum* species associated with anthracnose of jute in China using a muti-locus phylogenetic approach. In this study, we isolated 7 *Colletotrichum* strains representing 3 distinct taxa, 2 of which have not been previously associated with jute disease. Although this investigation is limited in the sampling scale and isolations obtained, it appears that jute may harbor more *Colletotrichum* species than previously expected.

The most striking finding of this study was the absence of the *C. gloeosporioides* that was previously reported to be one of the main causal agents of jute anthracnose. However, 2 members of the *C. gloeosporiodes* species complex were newly associated with jute anthracnose, *C. fructicola* (located in Youxi, Fujian province) and *C. siamense* (located in Nanning, Guangxi province). Although *C. fructicola* and *C. siamense* were isolated only from symptomatic stems, pathogenicity tests showed that both species can also cause anthracnose on jute leaves. This could indicate that *C. fructicola* and *C. siamense* begin their lifecycles as endophytes and grow into opportunistic pathogens^30^.

*Colletotrichum fructicola* was previously found to be an important pathogen on a variety of hosts^13^,^14^,^15^, and was also found as a leaf endophyte in several plants^13^,^31^. However, this is the first report *of C. fructicola* causing jute anthracnose. Similarly, *Colletotrichum siamense* is another species that had not been thought to cause anthracnose in jute in southeastern China. This species was originally isolated from coffee berries in Thailand, and was biologically and geographically diverse^15^,^29^. Pathogenicity tests showed that this species can cause disease of both the leaves and stems of jute. Interestingly, a recent study by Sharma *et al.* of *ApMat* sequence data recognized several clades within *C. siamense*, suggesting *C. siamense* may be a species complex^32^,^33^. Although the strain FAFU04 resembles the type strain of *C. siamense* (ICMP18642) with bootstrap support/posterior probability values of 100%/0.99 (Fig. 1), further collections and investigations need to be conducted to gain a better understanding of its phylogenetic relationships and infraspecific variation. *Colletotrichum corchorum-capsularis* (FAFU02, FAFU03, FAFU05, FAFU06 and FAFU07) produced curved conidia (Fig. 3), which have similarity to species in the *C. truncatum* species complex. Phylogenetic analysis showed that these 5 strains with curved conidia formed a distinct clade with 100% bootstrap support, indicating that they represent a distinct species. The morphological characteristics of these 5 strains were most closely related to those of *C. corchorum*, as they both produced colonies of same color and growth rate at 28 °C. However, the conidial length of the former is significantly (*P* < 0.05) longer (18.3–26.3 μm) than that from *C. corchorum* (12–25 μm)^18^. Thus, *Colletotrichum corchorum-capsularis* is introduced by this study to accommodate this species.

Pathogenicity tests showed that 3 species were pathogenic to jute leaves and stems, and the virulence was significantly different. *C. corchorum-capsularis* was the most virulent species with a mean incidence of disease of 83% on leaves, while *C. fructicola* and *C. siamense* showed mild virulence (Table 3). Symptom development may vary considerably with factors such as species, inoculation conditions, humidity, temperature, and the concentration of the inoculum^34^,^35^. Therefore, this result may not reflect the true virulence potential of these species. Additional research should be conducted to determine the virulence potential of *Colletotrichum* species in natural infections rather than artificial inoculations.

In the present study, we have combined morphological and molecular data to identify the species of *Colletotrichum* that cause disease of jute (*C. capsularis*) in the most important jute producing areas of China. The most important causal agent was *C. corchorum-capsularis*. *C. corchorum-capsularis* encompasses the most virulent strains and appears to be responsible for most jute anthracnose in China (Fujian, Henan and Zhejiang provinces). *C. corchorum-capsularis* shows phylogenetic divergence and is probably a species complex; further work with more discerning genes is required to characterize the new species. This is the first report to link *C. fructicola* and *C. siamense* to jute anthracnose. Both caused disease in Fujian and Guangxi provinces. Pathogenicity tests showed that both species could cause disease at similar frequencies. *C. gloeosporioides*, which is reported to be the main pathogen for jute anthracnose, was not found in this study, possibly because we did not survey in the whole vegetative period, and the collected strains were only from the infected stems.

## Additional Information

**How to cite this article**: Niu, X. *et al. Colletotrichum* species associated with jute (*Corchorus capsularis* L.) anthracnose in southeastern China. *Sci. Rep.* **6**, 25179; doi: 10.1038/srep25179 (2016).

## Figures and Tables

**Table 1:** Strains of *Colletotrichum* with details of culture collection, references and GenBank accessions of the sequences generated.

Species	Strains	References	GenBank accession Numbers					
			ACT	TUB2	CAL	GAPDH	GS	ITS
*C. aenigma*	ICMP 18608*	Weir *et al.*^15^	JX009443	JX010389	JX009683	JX010044	JX010078	JX010244
*C. aeschynomenes*	ICMP 17673*	Weir *et al.*^15^	JX009483	JX010392	JX009721	JX009930	JX010081	JX010176
*C. alatae*	ICMP 17919*	Weir *et al.*^15^	JX009471	JX010383	JX009738	JX009990	JX010065	JX010190
*C. alienum*	ICMP 12071*	Weir *et al.*^15^	JX009572	JX010411	JX009654	JX010028	JX010101	JX010251
*C. anthrisci*	CBS 125334*	Damm *et al.*^28^	GU227943	GU228139	–	GU228237	–	GU227845
	CBS 125335	Damm *et al.*^28^	GU227944	GU228140	–	GU228238	–	GU227846
*C. aotearoa*	ICMP 18537*	Weir *et al.*^15^	JX009564	JX010420	JX009611	JX010005	JX010113	JX010205
*C. asianum*	MFLUCC090233*	Prihastuti *et al.*^10^	FJ907424	FJ907439	FJ917506	FJ972576	JX010096	FJ972612
	MFLUCC090232	Prihastuti *et al.*^10^	FJ903188	FJ907434	FJ917501	FJ972571	FJ972586	FJ972605
*C. boninense*	MAFF305972*	Yang *et al.* 2011	HM582001	HM585421	HM582004	HM585386	–	HM585399
*C. brevisporum*	BCC 38876*	Noireung *et al.* 2012	JN050216	JN050244	–	JN050227	–	JN050238
*C. camelliae*	ICMP 10643	Weir *et al.*^15^	JX009540	JX010436	JX009630	JX009908	JX010119	JX010224
*C. circinans*	CBS 111.21	Damm *et al.*^28^	GU227952	GU228148	–	GU228246	–	GU227854
	CBS 221.81*	Damm *et al.*^28^	GU227953	GU228149	–	GU228247	–	GU227855
*C. clidemiae*	ICMP 18658*	Weir *et al.*^15^	JX009537	JX010438	JX009645	JX009989	JX010129	JX010265
***C. corchorum-capsularis***	**FAFU 02**	**this study**	–	**KT439340**	**KT439353**	**KT439360**	**KT439367**	**KT439374**
	**FAFU 03**	**this study**	**KT439347**	**KT439341**	**KT439354**	**KT439361**	**KT439368**	**KT439375**
	**FAFU 05**	**this study**	**KT439349**	**KT439343**	**KT439356**	**KT439363**	**KT439370**	**KT439377**
	**FAFU 06**	**this study**	**KT439350**	**KT439344**	**KT439356**	**KT439364**	**KT439371**	**KT439378**
	**FAFU 07**	**this study**	**KT439351**	**KT439345**	**KT439358**	**KT439365**	**KT439372**	**KT439379**
*C. cordylinicola*	ICMP 18579*	Weir *et al.*^15^	HM470235	JX010440	HM470238	JX009975	JX010122	JX010226
*C. curcumae*	IMI 288937*	Damm *et al.*^28^	GU227991	GU228187	–	GU228285	–	GU227893
*C. dematium*	CBS 125.25*	Damm *et al.*^28^	GU227917	GU228113	–	GU228211	–	GU227819
	CBS 125340	Damm *et al.*^28^	GU227918	GU228114	–	GU228212	–	GU227820
*C. fructi*	CBS 346.37*	Damm *et al.*^28^	GU227942	GU228138	–	GU228236	–	GU227844
*C. fructicola*	ICMP 18581*	Prihastuti *et al.*^10^	FJ907426	FJ907441	FJ917508	FJ972578	JX010095	FJ972603
	ICMP 18613	Weir *et al.*^15^	JX009491	JX010388	JX009675	JX009998	JX010077	JX010167
	ICMP 18727	Weir *et al.*^15^	JX009565	JX010394	JX009682	JX010035	JX010083	JX010179
	ICMP 18646	Rojas *et al.*^31^	JX009581	GU994470	JX009674	JX010032	JX010099	GU994372
	ICMP 17921*	Weir *et al.*^15^	JX009495	JX010400	JX009671	JX009923	JX010090	JX010181
	**FAFU 01**	**this study**	**KT439346**	**KT439339**	**KT439352**	**KT439359**	**KT439366**	**KT439373**
*C. gloeosporioides*	ICMP 17821*	Liu *et al.*^29^	JX009531	JX010445	JX009731	JX010056	JX010085	JX010152
	ICMP 12939	Weir *et al.*^15^	JX009462	–	JX009728	JX009931	–	JX010149
*C. jasminigenum*	MFLUCC 100273*	Weir *et al.*^15^	HM131508	HM153770	HM131494	HM131499	HM131504	HM131513
*C. kahawae subsp. ciggaro*	ICMP 18539*	Weir *et al.*^15^	JX009523	JX010434	JX009635	JX009966	JX010132	JX010230
*C. kahawae subsp. kahawae*	ICMP 17816*	Weir *et al.*^15^	JX009452	JX010444	JX009642	JX010012	JX010130	JX010231
*C. lindemuthianum*	CBS 151.28	Damm *et al.*^28^	GU227898	GU228094	–	GU228192	–	GU227800
*C. lineola*	CBS 125337*	Damm *et al.*^28^	GU227927	GU228123	–	GU228221	–	GU227829
	CBS 125339	Damm *et al.*^28^	GU227928	GU228124	–	GU228222	–	GU227830
*C. murrayae*	GZAAS5.09506*	Peng *et al.* 2012	JQ247657	JQ247644	JQ247596	JQ247609	JQ247621	JQ247623
	GZAAS5.09538	Peng *et al.* 2012	JQ247656	JQ247645	JQ247597	JQ247608	JQ247620	JQ247632
*C. musae*	CBS 116870*	Su *et al.* 2011	JX009433	HQ596280	JX009742	JX010050	JX010103	JX010146
	MFLUCC 100976	Su *et al.* 2011	HQ596285	HQ596281	HQ596296	HQ596300	HQ596289	HQ596293
*C. nupharicola*	ICMP 18187*	Weir *et al.*^15^	JX009437	JX010398	JX009663	JX009972	JX010088	JX010187
*C. psidii*	ICMP 19120*	Weir *et al.*^15^	JX009515	JX010443	JX009743	JX009967	JX010133	JX010219
*C. queenslandicum*	ICMP 1778*	Weir *et al.*^15^	JX009447	JX010414	JX009691	JX009934	JX010104	JX010276
*C. salsolae*	ICMP 19051*	Weir *et al.*^15^	JX009562	JX010403	JX009696	JX009916	JX010093	JX010242
*C. siamense*	MFLUCC090230*	Prihastuti *et al.*^10^	FJ907423	JX010404	FJ917505	JX009924	JX010094	JX010172
	MFLUCC090231	Prihastuti *et al.*^10^	FJ907422	FJ907437	FJ917504	FJ972574	FJ972597	FJ972614
	**FAFU 04**	**this study**	**KT439348**	**KT439342**	**KT439355**	**KT439362**	**KT439369**	**KT439376**
*C. siamense*	ICMP 19118*	Weir *et al.*^15^	HM131507	JX010415	JX009713	HM131497	JX010105	HM131511
	ICMP 18642*	Weir *et al.*^15^	GQ856775	JX010410	JX009709	JX010019	JX010100	JX010278
*C. thailandicum*	BCC 38879*	Noireung *et al.* 2012	JN050220	JN050248	–	JN050231	–	JN050242
*C. theobromicola*	ICMP 18649*	Rojas *et al.*^31^	JX009444	GU994477	JX009591	JX010006	JX010139	GU994360
	ICMP 17927	Weir *et al.*^15^	JX009516	JX010373	JX009592	JX010024	JX010064	JX010286
	ICMP 17957	Weir *et al.*^15^	JX009575	JX010380	JX009597	JX009962	JX010063	JX010289
*C. ti*	ICMP 4832*	Weir *et al.*^15^	JX009520	JX010442	JX009649	JX009952	JX010123	JX010269
*C. tropicale*	ICMP 18653*	Rojas *et al.*^31^	JX009489	JX010407	JX009719	JX010007	JX010097	JX010264
*C. tropicicola*	BCC 38877*	Noireung *et al.* 2012	JN050218	JN050246	–	JN050229	–	JN050240
*C. truncatum*	CBS 151.35*	Damm *et al.*^28^	GU227960	GU228156	–	GU228254	–	GU227862
	CBS 119189	Damm *et al.*^28^	GU227961	GU228157	–	GU228255	–	GU227863
	CBS 195.32	Damm *et al.*^28^	GU227963	GU228159	–	GU228257	–	GU227865
*C. viniferum*	GZAAS5.08601*	Peng *et al.*^14^	JN412795	JN412813	JQ309639	JN412798	JN412787	JN412804
	GZAAS5.08608	Peng *et al.*^14^	JN412793	JN412811	JN412782	JN412800	JN412784	JN412802
*C. xanthorrhoeae*	ICMP 17903*	Weir *et al.*^15^	JX009478	JX010448	JX009653	JX009927	JX010138	JX010261

ICMP, International Collection of Microorganisms from Plants (New Zealand); MFLUCC, Mae Fah Luang University Culture Collection (Thailand).

CBS, Centraalbureau voor Schimmelcultures (Netherlands); MAFF, Ministry of Agriculture, Forestry and Fisheries (Japan).

IMI, CABI Genetic Resource Collection (UK); GZAAS, Guizhou Academy of Agriculture Sciences (China);

FAFU, *Colletotrichum* strains collected in Fujian Agriculture and Forestry University (China); BCC, BIOTEC Culture Collection (Thailand);

*indicate the ex-type culures. New strains and accession numbers produced in this study are bold.

**Table 2:** Summary of morphological data of *Colletotrichum* isolates.

Species and isolates	Conidia			Appressoria		Growth rate (mm/day)*
	Length (μm)*	Width (μm)*	Shape	Length (μm)*	Width (μm)*	
*C. corchorum-capsularis* (FAFU02)	22.60 (±3.10)	3.62 (±0.62)	Curved	8.48 (±0.93)	7.26 (±1.25)	6.78 (±0.22)
*C. corchorum-capsularis* (FAFU03)	22.35 (±3.12)	3.25 (±0.50)	Curved	8.33 (±1.87)	7.23 (±1.36)	6.76 (±0.38)
*C. corchorum-capsularis* (FAFU05)	21.75 (±3.50)	3.50 (±0.50)	Curved	8.58 (±1.66)	7.25 (±1.32)	7.03 (±0.67)
*C. corchorum-capsularis* (FAFU06)	22.50 (±2.75)	3.50 (±0.50)	Curved	8.76 (±1.01)	7.35 (±0.67)	6.61 (±0.44)
*C. corchorum-capsularis* (FAFU07)	22.56 (±2.60)	3.37 (±0.62)	Curved	8.75 (±0.90)	7.36 (±0.63)	6.84 (±0.10)
*C. fructicola* (FAFU01)	11.21 (±2.63)	3.88 (±0.77)	Straight	8.25 (±0.99)	5.03 (±0.58)	6.86 (±0.30)
*C. siamense* (FAFU04)	11.91 (±2.89)	3.78 (±0.88)	Straight	8.75 (±1.75)	6.38 (±1.97)	9.02 (±0.34)

*indicates all figures given in Table 2 are mean values.

**Table 3:** Pathogenicity testing of *Colletotrichum* species from *C.capsularis*.

Species and isolates	Mean infection incidence (%)		Lesion diameter of stems (mm)	
	Leaves	Stems	Mean Length	Mean Width
*C. corchorum-capsularis* (FAFU02)	83	100	10.7 ± 1.70	6.4 ± 0.27
*C. fructicola* (FAFU01)	62	100	7.5 ± 0.36	3.5 ± 0.25
*C. siamense* (FAFU04)	58	100	6.7 ± 0.51	3.6 ± 0.38
control	0	0	–	–
